# The Dual Role of Vitamin K2 in “Bone-Vascular Crosstalk”: Opposite Effects on Bone Loss and Vascular Calcification

**DOI:** 10.3390/nu13041222

**Published:** 2021-04-07

**Authors:** Domitilla Mandatori, Letizia Pelusi, Valeria Schiavone, Caterina Pipino, Natalia Di Pietro, Assunta Pandolfi

**Affiliations:** Center for Advanced Studies and Technology-CAST (ex CeSI-MeT), StemTeCh Group, Department of Medical, Oral and Biotechnological Sciences, University “G. d’Annunzio” of Chieti-Pescara, 66100 Chieti, Italy; letizia.pelusi@unich.it (L.P.); valeria_95@libero.it (V.S.); c.pipino@unich.it (C.P.); natalia.dipietro@unich.it (N.D.P.); assunta.pandolfi@unich.it (A.P.)

**Keywords:** vitamin K2, menaquinone, calcium paradox, osteoporosis, vascular calcification

## Abstract

Osteoporosis (OP) and vascular calcification (VC) represent relevant health problems that frequently coexist in the elderly population. Traditionally, they have been considered independent processes, and mainly age-related. However, an increasing number of studies have reported their possible direct correlation, commonly defined as “bone-vascular crosstalk”. Vitamin K2 (VitK2), a family of several natural isoforms also known as menaquinones (MK), has recently received particular attention for its role in maintaining calcium homeostasis. In particular, VitK2 deficiency seems to be responsible of the so-called “calcium paradox” phenomenon, characterized by low calcium deposition in the bone and its accumulation in the vessel wall. Since these events may have important clinical consequences, and the role of VitK2 in bone-vascular crosstalk has only partially been explained, this review focuses on its effects on the bone and vascular system by providing a more recent literature update. Overall, the findings reported here propose the VitK2 family as natural bioactive molecules that could be able to play an important role in the prevention of bone loss and vascular calcification, thus encouraging further in-depth studies to achieve its use as a dietary food supplement.

## 1. Introduction

Osteoporosis (OP) is the most common bone disease that affects elderly men and women [1]. It is a metabolic skeletal disorder caused by an imbalance between bone formation and resorption, leading to a loss of bone mass and quality, skeletal structure deterioration and an increased risk of fractures [2,3]. OP is classified into a primary and secondary form with distinct etiological backgrounds. Type 1 (primary) is typical of postmenopausal women in whom the decrease in estrogenic levels is associated with an inflammatory state linked to an increase in osteoclast activity and a consequent imbalance of bone metabolism, whereas type 2 (secondary) occurs in both males and females, but its pathologic mechanism has only partially been clarified [4].

Vascular calcification (VC) is defined as the ectopic deposition of mineral matrix in the vessel wall. It occurs prevalently in aging and primary chronic conditions (hypertension, diabetes mellitus and chronic kidney disease), representing an important risk factor for cardiovascular morbidity and mortality [5,6,7,8]. Previously, the calcification of the vessel wall was known as a passive, degenerative and uncontrolled process caused only by the abnormal precipitation of calcium crystal in the vasculature [9,10]. Nowadays, a growing body of evidence suggests that it is an active, regulated event that shares similar characteristics with bone formation and metabolism. In particular, its discovery in the calcified vessel of bone-related proteins, bone-like structures and osteoblastic like-cells derived from vascular smooth muscle cells (VSMCs) has highlighted the active and cell-mediated nature of this vascular process [11,12,13,14,15,16].

Although OP and VC produce differing pathophysiological effects, their onsets frequently coexist in aging, representing one of the main public health problems with significant morbidity and mortality [17].

For many years, their coexistence was considered independent and only related to age [18], but several studies have provided support for a close link between bone and vascular health (Table 1) [19,20].

In this regard, many findings suggest that bone loss in OP may promote and increase the risk of cardiovascular events and vascular atherosclerosis. In the Framingham, Women’s Health Across the Nation (SWAN), Multi-Ethnic Study of Atherosclerosis (MESA) and Rotterdam studies, loss of bone mineral density (BMD) was associated with the development and progression of aortic calcification as well as with a higher risk of cardiovascular disease (CVD) mortality [21,22,23,24,25,26,27]. On the other hand, a direct correlation between VC and risk of bone fracture was also found. The MINOS study, for example, emphasized that men with aortic calcification present a major risk of bone fracture [28]. This was also found in healthy post-menopausal women with aortic calcification associated with lower BMD and increased risk of femur fractures (2.3-fold increase) [29].

Different hypotheses have been proposed to better explain the link between bone and vascular system, which is commonly referred to as “bone-vascular crosstalk”.

First, bone loss and vascular calcification share common risk factors, including smoking, physical activity, alcohol intake, Type 2 diabetes, menopause and hypertension. In addition, both are characterized by chronic low-grade inflammation and oxidative stress and by the involvement of bone morphogenetic proteins (BMP), osteoprotegerin, and parathyroid hormone, thus also suggesting common pathophysiologic mechanisms [19].

In this context, it is important to mention the role of the VitK2 family, lipid soluble compounds that play a pivotal role in the maintenance of calcium homeostasis [30]. Specifically, it is involved in the “calcium paradox”, a phenomenon in which a low calcium deposition in the bone tends to be associated with a parallel increase of calcium deposition in the vessel wall as a consequence of impaired calcium metabolism [31,32,33].

Given that the role of VitK2 in bone-vascular crosstalk and the “calcium paradox” has only been partially explained, this review aims to describe and summarize the most relevant knowledge concerning the nature of this vitamin, its molecular mechanism and clinical outcomes at bone and vascular level.

## 2. Vitamin K, a Family of Essential Fat-Soluble Compounds

Vitamin K is a family of essential fat-soluble compounds first identified in the early 1930s by the Danish biochemist Hendrik Dam during his research on cholesterol metabolism [34]. He observed that chicks fed a low-fat and sterols-free diet showed increased bleeding, which did not disappear when cholesterol was replaced in the diet [35]. Successively, Dam identified the “anti-haemorrhagic factor” in a fat-soluble compound that he named “Koagulation vitamin” (abbreviated vitamin K) to indicate its ability to clot blood [36].

Vitamin K naturally exists in two main forms: Vitamin K1 and Vitamin K2 [37]. Structurally both shared the central 2-methyl-1,4 naphthoquinone ring, named “menadione”, with a side chain on the menadione 3-carbon position [32].

**Figure 1:**
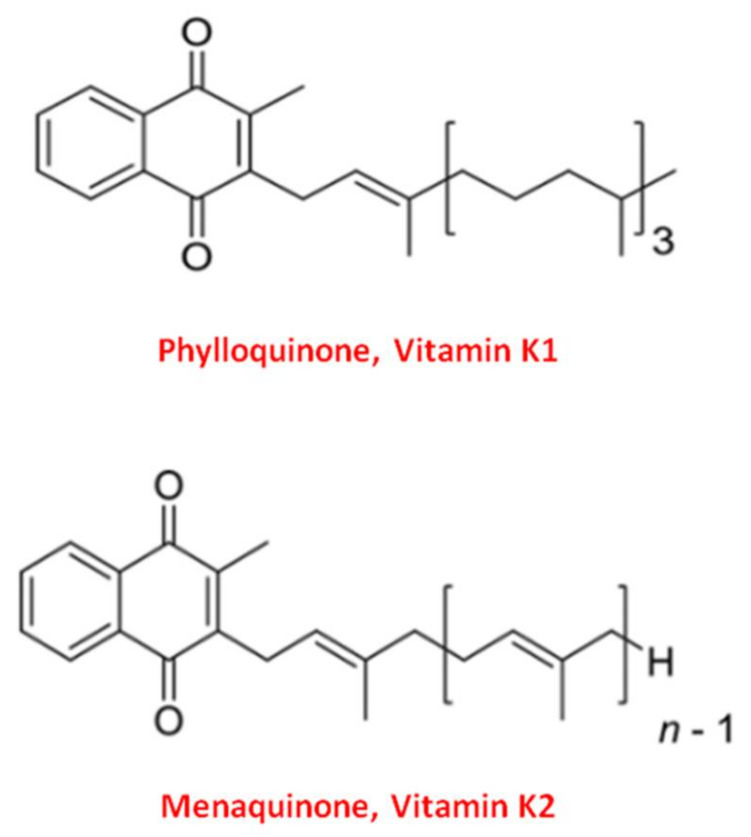
Molecular structure of the two main forms of Vitamin K. The upper structure represents Vitamin K1, also known as phylloquinone. The bottom structure is Vitamin K2, also known as menaquinone (MK).

Vitamin K1, or phylloquinone, contains a phytyl chain of four isoprenoid residues. In contrast, Vitamin K2 presents a side chain based on the repeating, from 4 to 13, of unsaturated isoprenoid units [38] (Figure 1).

All K-forms exert their biological function as cofactors for the Gamma-Glutamyl Carboxylase (GGCX), an enzyme which catalyzes the post-translational modification known as the “Vitamin K cycle” reaction [39]. More specifically, GGCX allows the conversion of the amino acid glutamate (Glu) into γ-carboxyglutamate (Gla) residues in at least another 14 specific proteins called “Vitamin K-dependent Proteins” (VKDPs), that, once activated, are able to bind calcium through their Gla residues [40].

Both Vitamin K1 and Vitamin K2 act as cofactors of GGCX in the “Vitamin K cycle”. However, Vitamin K1 triggers the activation of hepatic VKDPs implicated in the coagulation process (factor II, VII, IX and X). Whereas Vitamin K2 activates the VKDPs of extra-hepatic origin, such as Osteocalcin (OC) and Matrix Gla Protein (MGP) [37,41,42].

## 3. Vitamin K2 and Its Biomolecular Mechanisms of Action

The term VitK2 indicates a family of bioactive isoprenologs, also called “menaquinones” (MKs), which differ from each other with respect to the number of isoprenoid units in the side chain [32]. Thus, it is generally denoted as MK-n, where “n” (1–15) is the number of isoprenoid residues in the side chain [43,44,45]; for example, the isoforms menaquinone-4 (MK-4) and menaquinone-7 (MK-7) present four and seven isoprenoid units, respectively [43].

Most of the production of VitK2 in the human body take place at the intestinal level, where it is synthesized by intestinal bacteria of the genera Bacteroides, Lactococcus and Escherichia Coli [46,47]. However, the amount of VitK2-derived from intestinal bacteria is poorly absorbed and is not able to reach the optimal concentration required to exert the physiological functions [48,49]. Therefore, this vitamin should be supplemented daily with dietary sources such as animal-based foods (meat and egg yolk), bacterially fermented cheese, and the traditional Japanese dish called Natto, a fermented soybean in which the presence of *Bacillus Subtilis* reaches up to 1100 µg/100 g of VitK2 [50].

Regarding its metabolism, the various K2 isoforms show different bioavailability, and there is a direct correlation between their side chain length, lipophilicity, intestinal uptake and bioavailability in the human body [37,51].

As described in the previous paragraph, the compound members of VitK2 family are specific GGCX cofactors essential for the activation of extra-hepatic VKDPs. Specifically, through the activation of MPG and OC, VitK2 regulates the “calcium paradox” by reducing calcium deposition in the vessel wall and increasing it in the bone tissue, respectively. This results in the promotion of the bone mineralization process and a parallel inhibition of ectopic VC [38].

Osteocalcin, also known as bone γ-carboxyglutamate (Gla) protein or “Bone Gla Protein” (BGLP), was the first extra-hepatic VKDPsidentified, and represents the most abundant, non-collagenous protein in the mineralized bone matrix [52,53]. It is a secretory small peptide of 49 amino acids and 5.6 kDa [54,55] synthesized by osteoblasts and resealed into bone microenvironment in two circulating forms: carboxylated (cOC) and undercarboxylated (ucOC) [56].

**Figure 2:**
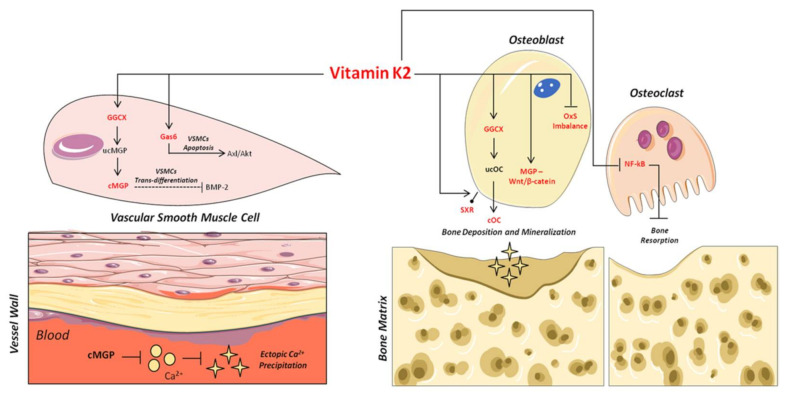
Mechanisms of action of VitK2 in “bone and vascular cross-talk”. At vascular level, VitK2, acting as cofactor for the enzyme GGCX, triggers the conversion of undercarboxylated MGP (ucMGP) in active carboxylated MGP (cMGP). The active cMGP could directly inhibit ectopic Ca2+ precipitation, but also VSMCst trans-differentiation through BMP-2. VitK2 can also inhibit VSMCs apoptosis through the Gas6/ AxL/Akt anti-apoptotic pathway. In bone tissue, VitK2 could promote osteoblasts proliferation and activity through MGP and Wnt/β-catenin pathway, control of oxidative stress (Ox-S) imbalance, via SXR receptor, and the well-established GGCX-dependent pathway. VitK2 may also exert a control of osteoclasts activities through the inhibition of NF-kB.

As shown in Figure 2, the carboxylated form plays an important role in the binding and precipitation of calcium-hydroxyapatite (Ca-HA), allowing bone matrix mineralization [57]. Once the mineralization process has been induced, cOC remains trapped in the bone matrix, and then it is released upon bone degradation into the circulation as ucOC [56,58]. Consequently, serum levels of cOC, ucOC and their ratio have to date been considered important biomarkers of bone turnover status, both in healthy and osteoporotic subjects [59,60].

In addition, ucOC also plays an important function as a bone-derived hormone able to enhance insulin secretion, sensitivity, energy expenditure and glucose homeostasis [61,62,63]. Thus, it was recently designated as a predictor and potential therapeutic target of several metabolic diseases, including diabetes [64,65].

Similarly, Matrix Gla Protein belongs to the family of extra-hepatic VKDPs, but it plays a significant role in the prevention of ectopic calcification in vascular system. It is a secretory protein of 14 kDa, 88 amino acids and 5 Glu residues in positions 2, 37, 41, 47 and 52 [37].

Once synthesized by VSMCs in the vessel wall, MGP undergoes two types of post-translational modifications: γ-glutamate carboxylation and the serine phosphorylation [66,67]. Serine phosphorylation, in positions 3, 6 and 9, is catalyzed by the “Golgi-localized enzyme casein kinase” [68]; its precise function is not clear, although recent studies suggest that it may be implicated in MGP secretion into the extracellular micro-environment [69]. On the contrary, γ-carboxylation is necessary for the biological activation of MGP as an inhibitor of ectopic mineralization in the vessel wall [70,71].

The central role of MGP in vascular health was first demonstrated in 1997 through the development of MGP knock-out (−/−) mice. All mice lacking MGP died within 8 weeks of birth due to massive arterial calcification [72]. Subsequently, it was also found in humans that a loss-of-function mutation in the MGP gene results in Keutel syndrome [73], a rare autosomal recessive disease characterized by ectopic calcification of soft tissues [74].

Based on this, several mechanisms have been proposed to explain the inhibitory role of carboxylated MGP (cMGP) on ectopic vascular mineralization. First of all, the ability of cMGP to directly inhibit calcium-phosphate crystal precipitation was demonstrated [75] (Figure 2). Furthermore, its role in the inhibition of VSMC trans-differentiation into osteoblastic-like cells [76] was highlighted. Indeed, MGP is able to inhibit the osteoblast trans-differentiation of VSMCs through the bone morphogenetic protein-2 (BMP-2) (Figure 2). The latter is one of the main osteogenic transcription factors, and has also been found in calcified atherosclerotic plaque and medial calcified lesions, where it exerts its function as an activator of VSMC osteogenic trans-differentiation [13,76,77,78]. In this regard, the active carboxylated form of MGP inhibits BMP-2 expression and its osteoinductive properties [79,80,81].

Based on these findings, and given that only the active MGP exerts the inhibitory role on ectopic mineralization, the uncarboxylated form (ucMGP) is currently recognized as a specific diagnostic marker of VC and cardiovascular clinical outcomes [82].

## 4. Vitamin K2 and Bone Health

Although several reports have underlined the important role of VitK2 in the maintenance of bone health, its exact function in bone metabolism has to date been poorly clarified [83,84]. This might be due to different shortcomings in the available studies, such as the limited numbers, structural heterogeneity and various isoforms of VitK2 used.

For this reason, in recent years, several randomized controlled trials (RCTs) have been conducted aiming to better investigate the effects of VitK2 supplementation in the prevention of bone loss and fracture in both healthy and osteoporotic patients (Table 2) [44].

Specifically, Kanellakis and collaborators [85], in a clinical trial of 219 osteoporotic postmenopausal women, found a significant increase in total BMD following one year of supplementation with VitK2 (100 μg/day). Thereafter, in a study that enrolled 244 healthy post-menopausal women, daily administration for three years of MK-7 (180 mcg/day) was associated with a decrease of bone loss and risk of vertebral fractures [86].

In support of this, a meta-analysis of 19 clinical trials including 6759 participants proved the effective role of VitK2 supplementation on BMD and risk of fractures in osteoporotic subjects [87]. Notably, these findings were recently confirmed by Mott and collaborators [88], who also described how, in osteoporotic post-menopausal women, treatment with this natural compound significantly reduced the level of ucOC and increased the active carboxylated form.

In this regard, it is well recognized that the primary mechanism by which VitK2 exerts its effects on bone health is through the γ-carboxylation of OC [93]. This has been supported by different bodies of evidence. In a placebo-controlled study with 55 adolescents, for instance, daily treatment with VitK2 (45 µg; MK-7) induced a significant decrease of ucOC with a significant parallel increase of cOC, resulting in an improvement of the bone mineralization process [89]. Subsequently, an increase of cOC serum concentration was also found in healthy adults (both male and females) subjected to daily vitamin administration [50,90,91,92]. This was similarly confirmed in post-menopausal women with osteoporotic fracture, with ucOC serum levels being comparable with young and healthy adults following MK-4 supplementation [88].

In addition to the clinical evidence reported above, the effects of VitK2 on bone metabolism have been investigated through pre-clinical animal studies.

In a model of osteoporotic ovariectomized (OVX) mice, Rangel and collaborators [94] demonstrated how this compound was able to improve BMD and bone formation markers, while decreasing bone resorption markers. Similar results were recently obtained in a model of osteopenic rats, which were characterized by an increase of bone formation and cOC serum levels following MK-4 treatment [95].

Based on the results obtained in in vivo models, several in vitro studies have been conducted to better understand the molecular mechanisms by which VitK2 acts in the bone system.

Remarkably, it was well established that it mainly acts on osteoblastic cells, improving their proliferation and differentiation and the function of bone matrix deposition through the aforementioned OC γ-carboxylation pathway [96].

However, it was also found that VitK2 enhances bone mineralization and decreases bone resorption in an OC γ-carboxylation-independent manner.

Indeed, a recent study demonstrated the involvement of MGP in the promotion of osteoblasts proliferation and activities through the Wnt/β-catenin signaling pathway [97].

Furthermore, it is known that oxidative stress plays a key role in the alteration of bone cell metabolism and bone disease development. In particular, the redox imbalance could trigger osteoblast and osteocyte apoptosis, inhibiting bone formation and mineralization. This may induce a shift of the bone anabolic process towards osteoclast activity, leading to an increase of bone loss [98]. In this regard, more recently Muszynska and collaborators [99] demonstrated that vitamin K2 compounds exert a protective effect on the protein pattern involved in bone formation and mineralization by using a model of the osteoblastic cell line in which oxidative stress was induced by hydrogen peroxide (H_2_O_2_).

In addition to this, the involvement of the nuclear steroid and xenobiotic receptor (SXR), a nuclear receptor which up-regulates the expression of the gene involved in osteoblast differentiation and bone matrix deposition, was found [100,101].

Finally, the anabolic effects of VitK2 at the bone level are also exerted through the regulation of different pathways implicated in osteoclast differentiation and activity; among these was the inhibition of the nuclear factor kappa-light-chain-enhancer of the activated B cells transcription factor (NF-kB) [102,103,104].

Given that these in vitro studies were mainly performed on differentiated bone cell cultures (osteoblasts and osteoclasts), we recently assessed the effects of VitK2 on osteogenic differentiation by using a model of human amniotic fluid primary mesenchymal stem cells (hAFMSCs). When these cells were cultured in a two-dimensional (2D) conventional system, we found that MK-4 treatment significantly improved their osteogenic commitment through the abovementioned γ-carboxylation-dependent pathway. In that study, to mimic the “bone remodeling unit” in vitro, we also co-cultured hAFMSCs with human monocytes (hMCs) as osteoclast precursors by using a three-dimensional (3D) dynamic system. Interestingly, we showed that MK-4 was able to promote hAFMSC osteogenic commitment and inhibit hMC osteoclast differentiation, thus promoting the formation of 3D bone aggregates potentially useful for tissue engineering applications in bone regenerative medicine [105].

## 5. Vitamin K2 and Vascular Health

The role of VitK2 in vascular health has been demonstrated by several studies emphasizing an inverse relation between its intake and the development of VC or consequent risk of cardiovascular events (Table 3) [106,107].

The first unexpected piece of evidence for a link between this molecule and VC was provided by some observational studies based on the use of vitamin K antagonists (VKAs). VKAs, such as Warfarin, are anticoagulants administrated to avoid thrombosis onset [37,108,109,110]. However, their use not only prevents the activation of clotting factors, but also the activation of extra-hepatic VKDPs (MGP and OC) [111]. This was demonstrated by several pre-clinical and clinical studies that showed that the use of VKAs was significantly associated with oxidative stress in VSMCs [112,113,114] and development of arterial calcification [49,115,116,117] in vessels, but also with loss of mineral density and increase of risk fractures in bone [118].

Based on such evidence, the relationship between VitK2 intake and the risk of cardiovascular events was further investigated in several clinical studies.

In the Rotterdam cohort, its daily supplementation (25 µg) was associated with a significant decrease in the risk of VC (by 52%), development of coronary heart disease (by 36%) and dying of heart disease (by 57%) [119]. Data from the Prospect-EPIC (European Prospective Investigation into Cancer and Nutrition) cohort study showed that a higher VitK2 intake was associated with a lower risk of Peripheral Arterial Disease (PAD) [120]. In a cross-sectional analysis among 564 post-menopausal women, MK use was correlated with reduced coronary calcification [106,121].

**Table 3:** Clinical evidence linking vitamin K2 supplementation and vascular health.

Study	Type of the Study	Number of Patients Enrolled	Key Findings
[120]	Meta-analysis of Prospect-EPIC cohort study	Healthy 16,057 women (49–70 years)	Menaquinone’s intake reduces the incidence of coronary heart disease
[121]	RCT	564 post-menopausal women	Menaquinone’s intake decreased coronary calcification
[122]	Prospective cohort	35,476 healthy subjects	Menaquinone’s dietary intake was not associated with reduced stroke risk
[106]	RCT	244 post-menopausal women	Vitamin K2 (MK-7; 180 μg/day) supplementation improves arterial stiffness
[123]	Prospective cohort study	7216 participants (Mediterranean population at high cardiovascular disease risk)	Vitamin K2 dietary intake was associated with a reduced risk of cardiovascular events and mortality
[124]	RCT	Patients with coronary artery disease (number not specified)	MK-7 (360 μg/day) supplementation arrested coronary artery calcification progression
[125]	Prospective cohort study	36,629 participants with PAD	Vitamin K2 intake was associated with a reduced risk of PAD
[126]	Prospective cohort study	2987 (Norwegian men and women)	Vitamin K2 intake was associated with a reduced risk of coronary artery disease
[127]	Prospective cohort study	33,289 participants from the EPIC-NL cohort	Higher intake Menaquinones was borderline significantly associated with lower CVD mortality
[128]	RCT	68 Type II diabetes and CVD patients	MK-7 (360 μg/day) was not associated with arterial calcification

Furthermore, VitK2 consumption was also positively associated with a reduction of mortality risk in a Mediterranean population characterized by high risk of CVD [123]. In a double-bind, randomized, placebo-controlled study among patients with coronary artery disease, the supplementation for 24 months with MK-7 slowed down or totally inhibited coronary artery calcification progression [124]. Similar results were obtained from a prospective cohort of peripheral arterial disease patients (36,629 participants), in which a higher vitamin dietary intake was associated with a reduced risk of PAD [125]. This was recently confirmed by Haugsgjerd and collaborators [126], who showed an inverse association with the risk of coronary heart disease in a prospective cohort study that enrolled 2987 Norwegian men and women.

However, despite the encouraging results mentioned above, no association has been found between VitK2 intake, reduced stroke risk [122], CVD mortality [127] and arterial calcification in Type 2 diabetes and CVD patients [128,129].

Regarding the molecular mechanism by which VitK2 might be involved in the regulation of vascular health, a growing amount of evidence emphasizes the strict link between VitK2 status, MGP and the development of cardiovascular events [130,131].

In this context, several observational studies have shown that the subjects with the highest levels of serum inactive ucMGP were characterized by increased VC, arterial stiffness, and higher risk of CVD [82,131,132,133]. Interestingly, a significant decrease of ucMGP was obtained with treatment with VitK2 [134,135,136,137]. However, it is important to underline that these results were mainly achieved in specific high-risk population groups, such as chronic kidney diseases (CKD), and in diabetic and hypertensive patients [42].

The inhibitory role of VitK2 was also investigated in some recent pre-clinical in vivo studies.

In a murine model of CKD-extraosseous calcification, supplementation with MK-7 (100 μg/g diet) inhibited the development of cardiovascular calcification via MGP pathway [71], whereas Wang and collaborators [138] found that this compound could inhibit the intimal calcification of the aortic artery in a high-fat diet in ApoE−/− mice model through the downregulation of the Toll-like receptor 2 (TLR2) and TLR4 expression. In addition, they confirmed these results in vitro using a cell line of VSMCs induced to the calcification process by β-sodium glycerophosphate [138].

In the same way, to better clarify the mechanism underlining the inhibitory role of VitK2, we recently performed an in vitro study with primary VSMCs exposed to β-sodium glycerophosphate.

Since hypertension is one of the main risk factors for cardiovascular calcification, VSMCs isolated from spontaneously hypertensive rats (SHR) were used as a model of cell vascular dysfunction [139]. Interestingly, we found that MK-4 reduced VC progression, preserving the contractile phenotype SHR-VSMCs. Specifically, we demonstrated for the first time that treatment with MK-4 was able to decrease the VC process through the inhibition of VSMC osteoblast trans-differentiation via MGP carboxylation, which triggered the inhibition of BMP-2 [140].

Finally, beyond the aforementioned role of VitK2 on VSMC osteoblast trans-differentiation through MGP, it is worth noting the involvement of the VKDPs, mainly expressed in the brain, and named Growth arrest-specific protein 6 (Gas6) [45]. Interestingly, since VSMC apoptosis represents another key event required for the development and progression of VC, it was hypothesized that the anti-apoptotic role of Gas6 VKDPs [141] may play a role in this scenario [8]. Indeed, both in rats in vivo that in rats-derived VSMCs in vitro, treatment with VitK2 was able to inhibit VSMC calcification, avoiding their apoptosis through the Gas6/AxL/Akt anti-apoptotic pathway [142,143].

## 6. Conclusions

In summary, although controversial results can still be found in the current literature [83,84,144], the evidence analyzed and reported in this review may support the idea that VitK2 can exert a relevant role in the maintenance of bone and vascular health.

With regard to bone disorders, its ability to reduce loss of BMD and fracture risk, as well as to improve bone quality, has been described by several clinical studies which, moreover, have confirmed that OC γ-carboxylation is the main mechanism of action through which this natural compound is able to improve bone health [88,90,92].

On the other hand, several bodies of clinical evidence suggest an analogous protective role of VitK2 at the vascular level, emphasizing a strict association between vitamin serum level, MGP γ-carboxylation levels, reduction of VSMCs osteogenic trans-differentiation and possibly the risk of cardiovascular events.

The VitK2 effects described in the regulation of the phenomenon named “bone-vascular crosstalk” occur through several molecular mechanisms, which are illustrated in Figure 2.

Specifically, at the vascular level, VitK2 acts as cofactor for GGCX, allowing the activation of MGP through its carboxylation. In turn, the active MGP carboxylated form may directly inhibit both the ectopic Ca2+ precipitation in the vessel wall and, in parallel, VSMC osteoblast trans-differentiation by inhibiting BMP-2 expression. Furthermore, VitK2 may inhibit VSMC calcification, avoiding their apoptosis through the Gas6/AxL/Akt anti-apoptotic pathway.

Simultaneously, in bone tissue, VitK2 is able to modulate several molecular pathways. In fact, in osteoblasts it can promote bone matrix deposition through the activation of the SXR receptor bone mineralization via GGCX and OC, as well as osteoblast proliferation and activity through the control of oxidative stress (Ox-S) imbalance and the involvement of MGP and the Wnt/β-catenin signaling pathway. Of note, VitK2 can also regulate the osteoclast function of bone resorption through the inhibition of NF-kB.

These interesting molecular effects exerted by VitK2 support the results of the pre-clinical and clinical studies reported here, implying that it can significantly promote bone and vascular health.

Therefore, VitK2 could be recommended as a natural bioactive compound potentially able to prevent and/or treat metabolic bone and vascular disease such as osteoporosis and vascular calcification.

## Figures and Tables

**Table 1:** Clinical evidence linking bone loss to vascular calcification.

Study	Name of the Study	Number of Patients Enrolled	Key Findings
[21]	Framingham Heart Study	364 women and 190 men (28–62 years old)	Bone loss was associated with progression of aortic calcification in women over 25 years
[22]	Women’s Health Across the Nation Study	90 women (45–58 years old)	Lower BMD was related to high aortic calcification
[23]	MESA Study	946 women (mean age 65.5 years old) and 963 men (mean age 64.1 years old)	Lower BMD was associated with greater coronary artery and abdominal aortic calcium score
[24]	Rotterdam Study	582 men and 694 women all >55 years old	BMD loss was significantly associated with higher follow-up coronary artery calcification

**Table 2:** Clinical evidence linking vitamin K2 supplementation and bone health.

Study	Type of the Study	Number of Patients Enrolled	Key Findings
[85]	RCT	219 post-menopausal women	BMD increase following one year of vitamin K2 supplementation (100 μg/day)
[86]	RCT	244 healthy post-menopausal women	Decrease bone loss following three years MK-7 supplement (180 μg/day)
[87]	Meta-analysis of 19 RCTs	6759 participants (post-menopausal women)	BMD improvement and low incidence of fracture in osteoporotic subjects following K2 treatment
[88]	Meta-analysis of 36 RCTs	11,122 participants (post-menopausal women)	Vitamin K2 treatment (MK-4: 45 mg/day) reduce fracture, increase cOC and decrease ucOC serum concentration
[89]	RCT	55 healthy children	8 weeks MK-7 supplementation increase cOC serum concentration
[90]	Non-placebo-controlled dose-examination study	55 healthy males	MK-4 supplementation (600 and 900 μg/day) decrease ucOC and increase cOC level respectively
[91]	RCT	48 healthy post-menopausal women	Serum ucOC concentrations were significantly lower following 6–12 months MK-4 treatment (1.5 mg/day)
[92]	RCT	60 postmenopausal women	MK-7 treatment (100 μg/day) significantly decrease ucOC and increase cOC/ucOC ratio

## Data Availability

Not applicable.
